# Positive association between sugar consumption and dental decay prevalence independent of oral hygiene in pre-school children: a longitudinal prospective study

**DOI:** 10.1093/pubmed/fdx184

**Published:** 2017-12-29

**Authors:** V Skafida, S Chambers

**Affiliations:** 1 Social Policy, School of Social and Political Sciences, University of Edinburgh, Edinburgh, UK; 2 MRC/CSO Social and Public Health Sciences Unit, University of Glasgow, Glasgow, UK

**Keywords:** children, dentistry and oral health, food and nutrition

## Abstract

**Background:**

Few studies explore how the longitudinal cumulative and combined effects of dietary habits and oral hygiene habits relate to dental decay in very young children.

**Methods:**

Using longitudinal survey data, logistic regression models were specified to predict dental decay by age 5. Predictor variables included questions on diet and oral hygiene from ages 2 to 5.

**Results:**

Compared to mainly eating meals, children who snacked all day but had no real meals had a higher chance of dental decay (odds ratios (OR) = 2.32). There was an incremental association between a decreasing frequency of toothbrushing at age 2 and higher chances of dental decay at age 5 (OR range from 1.39 to 2.17). Among children eating sweets or chocolate more frequently (once/day or more), toothbrushing more often (once/day; twice/day or more) reduced the chance of decay (OR of 2.11–2.26 compared to OR 3.60 for the least frequent brushing group). Compared to mothers in managerial and professional occupations, those who had never worked had children with a much higher chance of decay (OR = 3.47).

**Conclusion:**

This study has shown that toothbrushing can only in part attenuate the association between snacking and long term sugar consumption on dental decay outcomes in children under 5.

## Introduction

Sugar has received significant attention in the public health community and policy in recent years.^1^ Debate on the substantial health impacts of sugar consumption has led to some national governments introducing policy changes, such as taxes on foods high in sugar in response.^1,2^ In 2015, the WHO recommended that ideal added sugar consumption should be no more than 5% of total energy intake.^3^ Lowering sugar consumption has been put forward as a solution to soaring rates of diabetes and obesity.^4^ However, there has been less focus on the potential impact on dental decay, the most prevalent disease worldwide.^5^

Like other western countries, the dental health of the UK population has improved dramatically since the 1970s.^6^ Few adults now experience total tooth loss, and levels of decay amongst children in the UK compared with children across Europe are low.^6^ This is largely the result of improved dental care and prevention. Prevention has largely focused on improving oral hygiene practices rather than diet. These practices include toothbrushing twice daily with fluoride toothpaste, fluoride varnish application, and in some areas, water fluoridation.^7^ The evidence for the protective effect of fluoride in children is strong,^7,8^ however, there is conflicting evidence regarding whether oral hygiene habits, such as brushing with fluoride toothpaste, can attenuate the detrimental effects of high sugar diets in children.^9^

Within the UK, Scotland provides an interesting case study to understand the interplay between children’s diet, oral hygiene and dental health. Substantial resources have been invested in preventing dental decay in children through Childsmile,^10,11^ a national oral health programme. A universal and targeted programme, Childsmile aims to improve children’s oral hygiene through the promotion of toothbrushing and the delivery of fluoride varnish in nursery and school settings in areas of high deprivation. Since the programme’s introduction, decay levels have fallen in Scottish children.^12,13^ In more recent years, these falls have slowed, suggesting that improvements in oral hygiene may not attenuate other factors known to impact on decay, such as sugar consumption, which in pre-school children in Scotland is 15% of dietary intake,^14^ and even higher in areas of greatest deprivation.^15^ Social stratification in sugar consumption matches that of dental decay, which is experienced at a ratio of 2:1 among 5 year olds in areas of highest deprivation compared with those living in the areas of lowest deprivation.^12,13^

Yet, there is limited research on how the interplay between oral hygiene and diet affects dental decay in young children. UK studies^16,17^ have been limited to cross-sectional designs and showed mixed evidence that brushing teeth can attenuate the detrimental effects of a diet high in sugar. In a cross-sectional study, Masson *et al.*^15^ found that dental decay was linked to consumption of non-milk extrinsic sugars (NMES), but not total sugar in the diet of Scottish children. This association remained significant even for 3–17 year olds who reported brushing their teeth at least twice per day. The highest risk of dental decay was found among children who brushed their teeth once a day or less and were also in the highest tertile of NMES intake.^15^

There have been calls^9^ for more longitudinal studies on this topic. Longitudinal data can help unravel the cumulative and combined impact of dietary and oral hygiene practices over time. Also, previous work does not control adequately for a range of important sociodemographic variables, especially demographic variables which are collected for the mother, who often plays the leading role in decisions around children’s diets. In addition few studies focus on the under 5s,^15^ yet investigating links between diet and toothbrushing in very young children is essential given the need for early prevention and the focus in national oral health initiatives. Previous studies examining risk factors for caries longitudinally in pre-school children (some of whom were older than 5 years at follow up) have included data from relatively limited samples from the USA,^18^ Hong Kong,^19^ Germany^20^ and Finland.^21^ While each study suggested diet impacted on caries, none of the data were analysed using methods that allow for the determination of the extent to which oral hygiene habits can attenuate diet-related factors, or whether socioeconomic confounders attenuate both. Any additional impact of socioeconomic factors is particularly important to examine in a nationally representative sample where an oral health programme is in place with additional components targeting children living in areas of high deprivation. Our specific research questions ask:

- Is frequent consumption of sugar-rich foods associated with dental decay in children under 5?
- Do oral hygiene habits, specifically toothbrushing frequency, bedtime toothbrushing and frequency of dentist check-ups, attenuate any association between frequency of consumption of sugar-rich foods and dental decay in children under 5?
- Do sustained high levels of sugar consumption from year to year affect dental decay at age 5 differently depending on children’s oral hygiene practices?
- Does controlling for parental socioeconomic confounders attenuate any of the associations between frequency of consumption of sugar-rich foods, oral hygiene habits and decay?

## Methods

### Dataset description and justification

Growing Up in Scotland (GUS) is a longitudinal prospective study which provides information on dietary intake and frequency of snacking, on oral hygiene habits and dental decay, and on parental background variables for a large nationally representative sample of pre-school children in Scotland. It is the only longitudinal dataset in the UK to have questions on the aforementioned from different time points for children under 5. The cohort used in this analysis consisted, at the first survey, of 5217 babies born between 06/2004 and 05/2005. Babies were c. 10 months old at the time of the first sweep.^22^ Interviews were carried out in participants’ homes usually with the child’s mother. The stratified random sample draws on the Child Benefit Register. Appropriate sample weights were used for the analysis to adjust for non-random non-response bias, and for unequal probability of selection for some children. The official user guide for the first sweep of data describes the survey design in further detail.^22^ GUS received ethics approval by the Scotland ‘A’ MREC committee.

The main outcome, dental decay, is observed where children approach their fifth birthday (c. 58 months, fifth sweep of data). Relevant variables for the analysis from sweep 2 (children aged c. 22 months) were also used, denoted as ‘SW2’ in results tables. Although the survey runs annually, modules on diet and oral hygiene do not run every year. In total 3832 children had valid (non-missing) data at sweep 5 (from 5217 in sweep 1). Full attrition analysis is reported elsewhere.^23^ Our working sample consisted of 3770 children who had valid data at both sweep 2 and 5 on all variables explored in this study.

### Variables

#### Dental decay

A derived binary variable was created to capture dental decay in children aged just under 5. This was coded as 1 if the child’s parents reported that their child had tooth fillings, had a decayed tooth extracted, or had some or a lot of decay, and 0 otherwise.

#### Food consumption variables

Five variables on children’s eating habits were included in the analysis (Table 1). Where relevant, original ordinal responses were collapsed into two or three categories for the analysis. Complete details of original variable response categories can be found elsewhere.^23^ A question on whether children snacked or mainly ate at mealtimes was also controlled for, since prior research suggested that the frequency of food consumption is linked to dental decay.^9^ While dairy has a protective effect on teeth, we included a question on yoghurt in the analysis since many children’s yoghurts contain added sugar.^24^

**Table 1:** Descriptive statistics of key variables (total *N*: 3721)

Weighted data^a^	%	[95% CI]	*N*
Child has dental decay			
No	83.1	[81.4–84.6]	3164
Yes	16.9	[15.4–18.6]	557
How often does child drink soft drinks, not including diet or sugar-free drinks? (including diluting juice but not fresh fruit juice or water)			
Less than once/month or never	40.4	[37.8–43.1]	1515
Several times per month	59.6	[56.9–62.2]	2206
How often does child eat sweets or chocolates? (including only whole packets of sweets or a chocolates/chocolate bar, not individual sweets)			
Less than once/day	51.3	[49.0–53.6]	1998
Once/day or more	48.7	[46.4–51.0]	1723
Some children just have snacks all day while others wait for meals. How would you describe child? (SW2)			
Snacks all day and has no real meals	1.9	[1.5–2.4]	68
Snacks during the day but also has meals	75.4	[73.6–77.1]	2801
Does not snack much, just has meals	21.9	[20.3–23.7]	822
Other	0.8	[0.5–1.2]	30
If child is hungry between meals, what would child be most likely to eat? Yoghurts			
Not mentioned	32.3	[30.2–34.4]	1260
Mentioned	67.7	[65.6–69.8]	2461
If child is hungry between meals, what would child be most likely to eat? Fresh, dried or tinned fruit			
Not mentioned	32	[30.2–33.9]	1100
Mentioned	68	[66.1–69.8]	2621
If child is hungry between meals, what would child be most likely to eat? Sweets or chocolate			
Not mentioned	68.4	[66.6–70.1]	2552
Mentioned	31.6	[29.9–33.4]	1169
How easy or difficult do you find it to control the amount of sweets and sugary snacks or drinks that your child has?			
Very; fairly easy; neither easy nor difficult	80.9	[79.4–82.3]	3035
Fairly or very difficult	19.1	[17.7–20.6]	686
How often is a toothbrush used to clean child’s teeth?			
Twice/day or more	72.6	[71.0–74.1]	2727
Once/day	23.9	[22.6–25.3]	870
Less than once/day, rarely or not at all	3.5	[2.8–4.4]	124
Does child have to do any of the following at bedtime? Brush his/her teeth			
Always	88.6	[87.4–89.7]	3327
Usually	7.1	[6.2–8.0]	244
Sometimes or never	4.3	[3.7–5.1]	150
On average, how often does child attend a dentist for a routine check-up?			
Every 6 months or more often	83.7	[82.0–85.2]	3165
Every 12 months	7.2	[6.3–8.2]	266
Every 24 months or less often	1.8	[1.4–2.5]	62
Child never been to the dentist’s surgery, either for treatment or check-up	7.3	[6.0–8.9]	228
Maternal NS-SEC^26^			
Managerial and professional	48.2	[45.3–51.1]	2029
Intermediate	14.9	[13.7–16.2]	539
Small employers and own account holders	6.7	[5.8–7.8]	247
Lower supervisory and technical	8.6	[7.7–9.6]	295
Semi-routine and routine	19.5	[17.6–21.5]	566
Never worked	2.1	[1.5–3.0]	45
Maternal education			
Degree or equivalent	28.2	[25.7–30.9]	1229
Vocational qualifications	39.3	[37.6–41.1]	1471
Higher grade or equivalent	7.2	[6.3–8.2]	276
Standard grade	16.5	[14.8–18.4]	516
No qualifications	8.8	[7.4–10.4]	229
Mother’s age at birth of sample child^b^			
Under 20	7.6	[6.4–9.0]	169
20–29	40.9	[38.8–43.0]	1348
30–39	48.4	[46.1–50.7]	2049
40 or older	3.2	[2.6–3.8]	137
Ethnicity			
White	96.6	[94.6–97.9]	3626
Non-white	3.4	[2.1–5.4]	95
Gender			
Male	51.7	[49.8–53.5]	1903
Female	48.3	[46.5–50.2]	1818

^a^All *N* values are based on un-weighted data. 95% confidence intervals in brackets.

^b^Age inserted as interval variable in logistic regression models, here presented in banded form.

#### Oral hygiene variables

Three oral hygiene variables were introduced in the second model in the logistic regression analysis (Tables 1 and 2). Preliminary analyses showed that other survey questions, such as whether children had teeth brushed by a parent or brushed teeth themselves, or when children first used toothpaste and whether children used fluoride toothpaste, were not significant and have been omitted from the final analysis.

**Table 2:** Logistic regression analysis—models predict dental decay at age 5 (*N*: 3721)

Weighted data^a^	Model 1		Model 2		Model 3	
	OR	[95% CI]	OR	[95% CI]	OR	[95% CI]
How often does child drink soft drinks, not including diet or sugar-free drinks? Several times per month (Ref: less than once/month or never)	1.37^**^	[1.11,1.68]	1.34^**^	[1.09,1.64]	1.26^*^	[1.01,1.55]
How often does child eat sweets or chocolates? Once/day or more (Ref: less than once/day)	1.76^***^	[1.44,2.15]	1.74^***^	[1.42,2.12]	1.53^***^	[1.24,1.89]
Some children just have snacks all day while others wait for meals. How would you describe child? (SW2) (Ref: does not snack much, just has meals)						
Snacks all day and has no real meals	2.75^**^	[1.36,5.54]	2.67^**^	[1.28,5.57]	2.32^*^	[1.12,4.82]
Snacks during the day but also has meals	1.26	[0.94,1.70]	1.25	[0.93,1.68]	1.23	[0.91,1.66]
Other	0.43	[0.10,1.95]	0.45	[0.11,1.91]	0.40	[0.08,1.95]
If child is hungry between meals, what would child be most likely to eat? Yoghurts—mentioned (Ref: not mentioned)	1.39^*^	[1.06,1.83]	1.40^*^	[1.06,1.85]	1.27	[0.97,1.68]
If child is hungry between meals, what would child be most likely to eat? Fresh, dried or tinned fruit—mentioned (Ref: not mentioned)	0.68^***^	[0.56,0.83]	0.71^***^	[0.58,0.86]	0.90	[0.74,1.10]
If child is hungry between meals, what would child be most likely to eat? Sweets or chocolate—mentioned (Ref: not mentioned)	0.88	[0.73,1.06]	0.86	[0.71,1.04]	0.88	[0.72,1.06]
How easy or difficult do you find it to control the amount of sweets and sugary snacks or drinks that your child has? (SW2)						
Fairly or very difficult (Ref: very; fairly easy; neither easy nor difficult)	1.65^***^	[1.26,2.18]	1.68^***^	[1.26,2.24]	1.62^**^	[1.20,2.18]
How often is a toothbrush used to clean child teeth? (SW2) (Ref: twice/day or more)						
Once/day			1.42^**^	[1.13,1.80]	1.38^**^	[1.10,1.74]
Less than once/day, rarely or not at all			2.67^***^	[1.76,4.06]	2.16^**^	[1.37,3.40]
Does child have to do any of the following at bedtime?: Brush his/her teeth (Ref: always)						
Usually			1.38^*^	[1.00,1.91]	1.26	[0.90,1.76]
Sometimes or never			1.32	[0.85,2.07]	1.28	[0.82,1.99]
On average, how often does child attend a dentist for a routine check-up (Ref: every 6 months or more often)						
Every 12 months			0.59^*^	[0.37,0.96]	0.58^*^	[0.37,0.92]
Every 24 months or less often			0.57	[0.23,1.41]	0.42	[0.17,1.04]
Child never been to the dentist’s surgery, either for treatment or check-up			0.55^*^	[0.32,0.95]	0.39^**^	[0.22,0.71]
Maternal NS-SEC (Ref: managerial and professional)						
Intermediate					1.17	[0.83,1.66]
Small employers and own account holders					1.22	[0.80,1.88]
Lower supervisory and technical					1.24	[0.78,1.98]
Semi-routine and routine					1.95^***^	[1.44,2.64]
Never worked					3.47^**^	[1.56,7.74]
Maternal education (Ref: degree or equivalent)						
Vocational qualifications					1.91^***^	[1.37,2.67]
Higher grade or equivalent					1.68^*^	[1.07,2.63]
Standard grade					1.87^**^	[1.28,2.75]
No qualifications					2.29^***^	[1.47,3.58]
Mother’s age at birth of sample child (each additional year)					0.99	[0.97,1.02]
Mother’s ethnicity—non-white (Ref: white)					2.64^**^	[1.46,4.75]
Gender—female (Ref: male)					0.85	[0.69,1.05]
Nagelkerke pseudo *R*^2^	0.6		0.9		0.13	

^a^All *N* values are based on un-weighted data. Significance levels: *P < 0.05, **P < 0.01, ***P < 0.001. 95% Confidence intervals in brackets.

#### Background variables

The third and final model in the logistic regression analysis controlled for socioeconomic confounders based on the mother, and controlled for the child’s gender.

### Statistical analysis

Binary logistic regression models were specified where the binary outcome is coded so that models predict the incidence of dental decay. Independent variables were added in three steps. The first model controlled only for variables on eating habits; the second also controlled for oral hygiene habits; and in the third socioeconomic and background variables were added. Multicollinearity tests showed that none of the independent variables in the regression analyses reached the commonly used threshold of <0.200.^25^ Nagelkerke pseudo *R*^2^ is reported for each model as a rough indicator of how the predictive ability of the models changes with each set of added variables. Changes in odds ratios (OR) and significance values for select variables from one model to the next provide some indication of whether associations between select predictors and the outcome are being fully or partially explained by subsequently added variables. Interaction effects between sugar consumption at ages 2 and 5 and dental decay at age 5, explored for different toothbrushing habits are shown in Table 3. Stata version 14.1 was used for all analyses.

**Table 3:** Logistic regression—dental decay as predicted by longitudinal sugar consumption by different toothbrushing frequencies^a^

	Toothbrush used less than once/day		Toothbrush used once/day		Toothbrush used twice/day or more	
	OR	[95% CI]	OR	[95% CI]	OR	[95% CI]
How often does child eat sweets or chocolates?						
*Ref: Less than once/day—SW2 and SW5*						
Once/day or more often— SW2 and SW5	3.60^*^	[1.11,11.68]	2.11^**^	[1.28,3.49]	2.26^***^	[1.63,3.15]
Increased frequency from SW2 to SW5	1.31	[0.26,6.51]	1.34	[0.73,2.46]	1.78^**^	[1.24,2.56]
Decreased frequency from SW2 to SW5	1.01	[0.21,4.86]	1.33	[0.67,2.63]	2.18^***^	[1.46,3.25]
*N*	119		832		2632	
Nagelkerke pseudo R^2^	0.29		0.16		0.11	

Exponentiated coefficients; 95% confidence intervals in brackets.

^*^P < 0.05, ^**^P < 0.01, ^***^P < 0.001

^a^Models adjusted for socioeconomic confounders: Maternal NS-SEC, education, ethnicity and age at birth of sample child; child gender.

## Results

### Eating habits

Three of the six food consumption variables remained significantly associated with dental decay after controlling for oral hygiene and socioeconomic confounders. Children were significantly more likely to have dental decay by age 5 if they consumed soft drinks more frequently (OR = 1.24) and if they ate sweets or chocolates once per day or more often (OR = 1.56). Compared to children who at age 2 mainly ate meals and did not snack much, those who snacked all day but had no real meals had a higher chance of dental decay (OR = 2.32), which was only partly explained by socioeconomic factors. Children whose parents reported when children were aged 2 that it was difficult to control the amount of sweets and sugary snacks eaten were also more likely to have experienced dental decay by age 5 (OR = 1.68). Significant associations between fruit consumption and dental decay, and between yoghurt consumption and dental decay in models 1 and 2, were fully explained by controlling for socioeconomic confounders in Model 3.

### Oral hygiene

Children who at age 2 were using a toothbrush less often were more likely to have dental decay at age 5, and there was an incremental association between a decreasing frequency of toothbrushing and higher chances of dental decay (OR range from 1.39 to 2.17). For this variable, the association between using a toothbrush ‘Less than once/day, rarely or not at all’ with dental decay was partly explained by controlling for socioeconomic factors, but remained large and significant (OR changing from 2.68 to 2.17). Going for less frequent dental check-ups was associated with a lower (OR = 0.39) chance of dental decay, since dental decay would be diagnosed and identified at the dentist. Introducing the oral hygiene variables in Model 2 did not drastically alter the OR of the eating habits variables in Model 1, though a more detailed analysis of the interaction effects between oral hygiene and diet is explored below.

### Longitudinal patterns or sugar consumption

Table 3 suggests that among children who brushed less than once/day, those who persistently consumed sweets and chocolate more frequently across time points also had a much higher chance of dental decay compared to those who consistently ate such foods less often at both ages 2 and 5 (OR = 3.60). This suggests a cumulatively higher risk of dental decay for children who both consume higher sugar containing foods and who also brush less often, even when controlling for confounders.

Looking at trends in children’s frequency of consumption of sweets or chocolates in relation to dental decay, suggested that those less likely to have dental decay by age 5 were children who consistently ate sweets and chocolates less often at both ages 2 and 5, and this was the case both for children who brushed their teeth once/day and those who brushed twice/day or more often. For children eating sweets or chocolate more frequently (once/day or more), toothbrushing more often (either once or twice/day) attenuated the impact on decay (OR of 2.11–2.26 compared to OR 3.60 for the least frequent brushing).

### Socioeconomic confounders

Children in homes from more disadvantaged backgrounds, and non-white ethnic groups were far more likely to experience dental decay. Compared to mothers in managerial and professional occupations, those who had never worked had children with a much higher chance of decay (OR = 3.47). Children of mothers from non-white ethnic backgrounds were far more likely to experience decay (OR = 2.61).

## Discussion

### Main findings of this study

This study addressed a gap in understanding whether toothbrushing attenuates the impact of dietary sugars on tooth decay in pre-school children, and whether there are cumulative effects on dental decay for poor dietary habits which are sustained over time in the early years. The main findings were that frequent consumption of sugar-rich foods was associated with dental decay in children under 5. Lack of parental control over the amount of sweets or chocolate that children consume also predicted dental decay, controlling for confounders. In Model 1, eating yoghurts between meals increased the likelihood of decay, whilst eating fruit reduced the likelihood. Controlling for oral hygiene habits attenuated these associations to an extent, but not completely. Brushing less than twice per day was associated with an increased likelihood of tooth decay. Socioeconomic confounders also partly explained these associations, but not fully. Children from lower socioeconomic backgrounds were far more likely to experience decay, as were children of non-white mothers. The large sizes of the coefficients for the socioeconomic factors suggest that a large part of the dental decay is not explained by either the dietary nor the oral hygiene variables in this dataset. The longitudinal analysis highlighted that toothbrushing did not reduce decay for those children with infrequent consumption of sweets and chocolates at ages 2 and 5. However, for children who ate sweets at least once/day or more, toothbrushing could reduce the chances of decay. Those most likely to have decay at age 5 were children who consistently eat sugary foods more often at both ages 2 and 5, and who also brushed their teeth the least.

### What is already known

Previous studies in this area were limited and contradictory.^9^ The results from this study are in line with Hinds and Gregory^16^ and Masson *et al.*^15^ who found that toothbrushing does not fully control for the impact of diet on decay. In line with other longitudinal studies,^18–21^ our results highlight that dietary habits in the early years can have a significantly detrimental impact on children’s decay outcomes by age 5. Our longitudinal results also highlight that for children who are frequent consumers of sweet foods, toothbrushing at least daily can partially attenuate the impact of sugar on decay.

### What this study adds

Our results indicate that consumption of soft drinks, sweets and chocolates should be reduced to protect against dental decay, however, there are also changes required in relation to dietary practices more generally. Parents who reported feeling less in control of children’s sweet-food intake were more likely to have children with decay. It is unclear whether lack of control relates to children being in childcare, or issues relating to control and authoritative parenting styles more generally which can correlate with dental decay.^27^ Snacking habits was the variable most strongly associated with decay, with children who snack all day without eating meals having twice the odds of decay as those who snacked less. The results on snacking were consistent with other studies.^28–30^ In Scotland, parents are advised to limit sugary foods to mealtimes, however, our results suggest that snacking generally may be detrimental to children’s teeth. This is an area where oral health programmes could strengthen their impact.

The effect sizes of the socioeconomic confounders overshadowed most of the effect sizes of the dietary and oral hygiene variables, suggesting that ultimately parental socioeconomic background explain more of the difference in children’s dental decay than do either of the other two categories of variables more directly linked to tooth decay in physiological terms. This could be because socioeconomic variables are indirectly picking up practices related to diet and oral hygiene not appropriately captured in the survey or in the questions controlled for in this analysis. Nevertheless, it is a reminder that even with Childsmile, which specifically aims to reduce inequalities in children’s dental decay, and has targeted components, it remains an ongoing challenge to reduce social patterning in dental health outcomes.^12,13^

### Limitations of this study

This study used a large representative cohort to examine an under-researched area of child health where significant health inequalities remain. The data offered longitudinal insights into the links between diet, oral hygiene and dental decay. The main study limitations relate to the study measures. Decay measures were based on parental recall of children’s decay experience, and were also reliant on children having attended a dental examination. Around 7% of children had never been for a dental examination, which explains in part the tautological finding that dentist visits were associated with a greater likelihood of decay. The food frequency measures have also not been validated against gold standard weighed dietary measures, and were based solely on parental perceptions of what the child ate, and are as such subject to recall and reporting bias. Finally, questions on toothbrushing may be indirectly measuring the latent general approach parents adopt in taking care of children’s teeth, i.e. early prevention.

## Conclusion

This study has shown that toothbrushing can only in part reduce the impact of sugar consumption and snacking on dental decay outcomes in children under 5. Huge progress has been made around improving oral hygiene in the UK population, however, the same progress has not been seen in terms of sugar intake. Diets low in sugar, and particularly reduced sugar-snacking, must continue to be promoted to reduce dental decay in children. Policy measures tailored to the socially stratified nature of dental decay, which move beyond the promotion of specific protective behaviours and address structural determinants of dental health would be welcome.
